# Emerging risk of *Dirofilaria* spp. infection in Northeastern Europe: high prevalence of *Dirofilaria repens* in sled dog kennels from the Baltic countries

**DOI:** 10.1038/s41598-020-80208-1

**Published:** 2021-01-13

**Authors:** Mustafa Alsarraf, Viktoria Levytska, Ewa J. Mierzejewska, Vasyl Poliukhovych, Anna Rodo, Mohammed Alsarraf, Dziyana Kavalevich, Dorota Dwużnik-Szarek, Jerzy M. Behnke, Anna Bajer

**Affiliations:** 1 Department of Eco-Epidemiology of Parasitic Diseases, Institute of Developmental Biology and Biomedical Sciences, Faculty of Biology, University of Warsaw, 1 Miecznikowa Street, 02-096 Warsaw, Poland; 2 Department of Infection and Invasive Diseases, Faculty of Veterinary Medicine and Technology in Animal Husbandry, State Agrarian and Engineering University in Podilia, 13 Shevcnenka Street, Kamianets-Podilskyi, 32300 Ukraine; 3 Department of Pathology and Veterinary Diagnostics, Warsaw University of Life Sciences-SGGW, 159c Nowoursynowska Street, 02-766 Warsaw, Poland; 4 School of Life Sciences, University Park, University of Nottingham, Nottingham, NG7 2RD UK

**Keywords:** Infectious diseases, Climate-change ecology

## Abstract

Dirofilariasis is a fast-spreading disease of dogs and humans in Europe. We investigated whether *Dirofilaria* spp. have spread northwards in Europe, invading the Baltic countries. Altogether, 424 blood samples were collected from eight countries in the period 2017–2019, including 227 samples from sled dogs and 197 samples from other dogs. PCR amplification and sequencing were conducted employing three genetic markers (mitochondrial [mt] 12S rDNA, mt cytochrome c oxidase subunit I [COI] gene and mt dehydrogenase subunit I [NAD1] gene). The SNAP test (IDEXX) for detection of *D. immitis* infections was also implemented. The DNA of *D. repens* was detected in 59 of 424 dogs (prevalence 13.9%). *D. repens* was found in sled dogs from Lithuania, Latvia, Poland and Belarus. Only one dog from Estonia was infected, apparently an imported case. The highest prevalence was recorded in Lithuania (38%). Among pet dogs from the Ukraine, six dogs tested positive (3.8%). Our study has revealed a high prevalence of *D. repens* infections in Lithuania and Latvia, but no evidence for spread of the heartworm *D. immitis*. We conclude that sled dog kennels constitute hot spots for *D. repens* transmission.

## Introduction

Nematodes of the genus *Dirofilaria* are parasites vectored by mosquitoes, and domestic dogs constitute the main reservoir for both *Dirofilaria immitis* (heartworm) and *Dirofilaria repens*^1–5^. The list of mosquito species able to transmit filariae grows longer each year, consisting of both endemic species and invasive species of mosquitoes that are spreading into and within Europe due to climate change^1,3,4^. Global warming in particular, has created conditions favouring the development of infective larvae in mosquitoes, and facilitated the recent spread of *Dirofilaria* spp. to Central Europe. *Dirofilaria repens* is now endemic in many countries in the region (Poland, Ukraine, Germany, Austria, Hungary, Netherlands)^6–17^, and is currently considered to be one of the fast spreading zoonoses in Central, Eastern and Northern Europe^2,4,18–21^. Not surprisingly, the number of published human cases is increasing also with reports on unusual localization of *D. repens* in human hosts^22–28^.

In contrast less data are available on the occurrence of *D. immitis* in Europe^2^, although this species is considered to be endemic in some European countries (Romania, Serbia, Slovenia, Slovakia, Czech Republic)^29–33^ and recent reports have indicated the presence of *D. immitis* in Poland and the Baltic countries^34–36^. However, the region encompassing the Baltic (Lithuania, Latvia, Estonia) and Nordic countries has been studied less extensively than elsewhere in Europe^37–39^ and this region in Northern Europe is likely to be a new area for expansion of the range of *Dirofilaria* spp. The present study was carried out to determine the current prevalence of *Dirofilaria* spp. especially in the Baltic States, but also in some neighbouring countries in this region of Europe, by a systematic survey of sled dog kennels.

We focused on sled dogs because they are particularly prone to vector borne infections through their participation in training sessions and racing events, the majority of which take place in forest areas where ticks and mosquitoes are abundant. Moreover, these dogs are usually housed in kennels where there may be an additional high risk of vector challenge. Sled dogs are frequently translocated to various racing sites, often distant from their home kennels, but mostly in the autumn and winter, outside the period of *Dirofilaria* transmission. However, adult dogs are traded, exchanged between kennels, and can be imported from endemic countries to kennels in non-endemic regions. For these reasons, sled dogs constitute a useful sentinel host for monitoring changes in local prevalence of these filarial parasites. In non-endemic countries, knowledge and experience of mosquito-borne helminths may be inadequate and prophylaxis not frequently implemented^34,37,39^. In the case of *D. repens*, long-term asymptomatic infections may occur in dogs, constituting a constant source of infection for new generations of mosquitoes (for a period of 2–5 years^5,40,41^). Consequently, failure to identify infection in translocated dogs and/or to implement appropriate treatment, will result in dogs carrying *Dirofilaria* spp. serving as sources of microfilariae for local mosquitoes.

With this background, we hypothesize that sled dog kennels constitute new important foci for *Dirofilaria* spp. because of the suitable conditions for transmission in kennels, high dog density and importation of infected dogs from endemic countries. The specific aims of the current study were: (1) to determine the prevalence of *D. repens* infection in sled dog kennels from the Baltic countries and other countries in the region; (2) to analyze factors likely to influence prevalence of infection (i.e. life history, origin, sex and age of dogs); (3) where data were available, to compare prevalence in other countries, including endemic areas (Poland, Ukraine); and finally, (4) to assess the prevalence of *D. immitis* in sled dogs in the Baltic countries and in Poland, in case the expansion of *D. repens* has concealed an underlying, concurrent expansion also of this species.

## Materials and methods

### Ethics approval and consent to participate

Since the study was carried out on blood samples provided voluntarily by dog owners (including the director and veterinary services of a local dog shelter), no ethical approval/ license was required for our study (as per Resolution on the protection of animals used for scientific or educational purposes, 15th January 2015 [Dz. U. 2015 position 266] Chapter 1, Paragraph 1.2.1). The owners of dogs involved in this study were informed about the aims of the study, provided oral consent and contact information to obtain the results of testing. The director and veterinary services of a local dog shelter (Błędowo, Poland) were informed about the results of *Dirofilaria* testing of the dogs under their care.

### Collection of samples

Blood samples from dogs were collected in summer-autumn 2017 and in then again in the same seasons in 2019 (Table 1). Altogether, 424 samples of blood were collected into EDTA-covered vials, from dogs from eight countries, including 227 samples from various breeds of sled dogs (and dogs living in sled dog kennels; Supplementary Table 1), and 197 samples from other dogs (Table 1). In Poland, the other dogs comprised a diverse group of healthy dogs presenting no symptoms (owned pedigree dogs and mixed breed dogs from a shelter) from a single location (Błędowo near Nowy Dwór Mazowiecki, Central Poland). In Western Ukraine, samples were derived from a diverse group of dogs attending the Fauna-Service veterinary clinic (Kamianets-Podilskyi, Khmelnytsky Region) and the Vet House (Vinnytsia) for non-filarial-related concerns.

**Table 1:** Prevalence of *Dirofilaria repens* (molecular detection) in dogs by country, year of study and sled dog status.

	Year of study	Country								Sled dogs versus other dogs
			Baltic countries							
		Poland% infected (n/N)	Lithuania% infected (n/N)	Latvia% infected (n/N)	Estonia% infected (n/N)	Finland% infected (n/N)	Belarus% infected (n/N)	Russia% infected (n/N)	Ukraine% infected (n/N)	
Sled dogs	2017	12.5% (2/16)	58.1% (18/31)	31.3% (5/16)	0% (0/2)	0% (0/1)	60% (3/5)	nd	nd	39.4% (28/71)
	2019	12% (6/50)	23.9% (11/46)	8.3% (2/24)	5% (1/20)	0% (0/5)	nd	0% (0/11)	nd	12.8% (20/156)
	Total	12.1% (8/66)	37.7% (29/77)	17.5% (7/40)	4.5% (1/22)	0% (0/6)	60% (3/5)	0% (0/11)	nd	21.2% (48/227)
Other dogs	2019	11.9% (5/42)	nd	nd	nd	nd	nd	nd	3.9% (6/155)	5.6% (11/197)
Total	Total	12.0% (13/108)	37.7% (29/77)	17.5% (7/40)	4.5% (1/22)	0% (0/6)	60% (3/5)	0% (0/11)	3.9% (6/155)	13.9% (59/424)

n: number of positive dogs; N: number of examined dogs; nd: not done.

Sled dogs were sampled in their kennels or during sled dog races, approximately one ml of blood being taken from the cephalic vein by licensed veterinarians. Basic data on a sampled dog were noted, including breed, age, sex and health status (i.e. activity level, appetite, any health or stamina problems, any skin abnormalities). In the most affected kennels in Lithuania, the history of dog importation from endemic countries was also recorded. In 2019, sampling was expanded and in addition to blood collected into EDTA and blood smears, we carried out also SNAP tests for *D. immitis *in situ.

For a subset of 168 dogs from Lithuania, Latvia, Estonia (all sled dogs) and Poland (sled dogs and other dogs from Błędowo) sampled at their kennels (11 in 2017 and 157 in 2019), two thin blood smears were prepared directly after sampling, then stained with Hemacolor stain and inspected under microscopy (at 100 × and 200 × magnification) for the presence of microfilariae. A subset of 167 blood samples from the same kennels visited in 2019 were tested for circulating *D. immitis* antigens by the commercially available SNAP test (IDEXX Laboratories Inc., Maine, USA), performed accordingly to the manufacturer’s instructions.

In 2019, 16 of the dogs originally sampled in 2017 from two sled dog kennels in Lithuania (including six that were negative and ten that were positive in 2017) were re-sampled, enabling comparison of infection status and assessment of the success of treatment with ivermectin that had been administrated orally in 2017, but only to the *D. repens*-positive dogs.

### Molecular detection of *Dirofilaria repens*

Genomic DNA was extracted from EDTA-preserved blood samples using the DNAeasy Blood & Tissue kit (Qiagen, USA) and stored at a temperature of -20 °C.

Molecular detection of *D. repens* was performed by amplification and sequencing of three species-specific markers. A combination of *D. repens* specific primers (12SF and 12SR2) extracted from a multiplex PCR developed by Gioia et al.^42^ was used to amplify the mitochondrial *12S rRNA* gene fragment (327 bp) of *D. repens.*

Two sets of species-specific primers were designed based on the recently published mitochondrial genome of *D. repens* (KX265047)^43^. A fragment of about 800 bp of the cytochrome c oxidase subunit I (COI) gene was amplified using primers ctc2F (5′-GTGAATGTGTTTTGTTTTTGAC-3′) and ctc2R (5′-CCTTACTAATAACCTTTCAATGAA -3′). A gene fragment of dehydrogenase subunit I (NAD1), about 516 bp long, was amplified using primers nadF (5′-TTGTAGTATGGTAGAGGTAAGG-3′) and nadR (5′-TGAACACAAGAACGAATACC-3′).

For screening of samples using 12S rDNA, the PCR reactions were performed in a volume of 20 μl, including 1 × PCR Dream Taq Green buffer, 1U Dream Taq polymerase (Thermo Fisher Scientific, Waltham, Massachusetts USA), 0.33 mM dNTPs, 1 μM of each primer and 2 μl of the extracted DNA sample.

To obtain good quality PCR products for sequencing (COI and NAD1 gene fragments) we used Promega GoTaq G2 Hot Start polymerase (Promega, Wisconsin, USA). Positive control constituted DNA of *D. repens* obtained from our previous study^44^. Negative controls were performed with nuclease-free distilled water, in the absence of template DNA.

For amplification of 12S rDNA, PCR reactions were carried out in the following cycling conditions: primary denaturation at 94 °C for 3 min, then 40 cycles of denaturation at 94 °C for 45 s, annealing at 58 °C for 45 s, and elongation at 72 °C for 45 s, followed by final elongation at 72 °C for 7 min and a hold step at 4 °C. For amplification of the COI and NAD1 gene fragments, the conditions were: primary denaturation at 95 °C for 5 min, then 40 cycles of denaturation at 95 °C for 45 s, annealing at 61 °C for 1 min, and elongation at 72 °C for 1 min, followed by final elongation at 72 °C for 7 min and a hold step at 4 °C.

Amplicons were visualized with Midori Green stain (Nippon Genetics Europe GmbH, Germany) following electrophoresis in 1.5% agarose gels. Selected amplicons were purified and sequenced in both directions by a private company (Genomed S.A., Warsaw Poland). DNA sequence alignments were conducted using MEGA version 6.0 and Codon Code Aligner. The resulting consensus sequences were compared with sequences deposited in GenBank NCBI^40,44^.

Selected *D. repens* sequences originating from different countries have been deposited in the GenBank database under the accession numbers: COI: MW206364- MW206376; NAD1: MW311373- MW311374; MW327622-MW327639.

### Statistical analyses

For the analysis of prevalence (% infected), we applied maximum likelihood techniques based on log linear analysis of contingency tables in the IBM SPSS v. 26 software package (IBM Corporation). Country of dog origin (1–8), age class (three levels: class 1 encompassing dogs up to two years of age; class 2—dogs more than 2 years old and up to 8 years of age; class 3—dogs older than 8 years; Supplementary Table 1), sex of dogs (males and females), year of study (two levels: 2017 and 2019) and sled dog status (coded 0 for other dogs, 1 for sled dogs) were used as factors in models with the presence or absence of *D. repens* considered as a binary factor (0, 1) and referred to as infection. For each level of analysis in turn, beginning with the most complex model, involving all possible main effects and interactions, those combinations that did not contribute significantly to explaining variation in the data were eliminated in a stepwise fashion beginning with the highest level interaction (backward selection procedure) as applied in our earlier papers^40,44^. A minimum sufficient model was then obtained, for which the likelihood ratio of chi-square was not significant, indicating that the model was sufficient in explaining the data. The importance of each term in interactions involving infection in the final model was assessed by the probability that its exclusion would alter the model significantly and these values are given in the text, assessed by a likelihood ratio test between nested models with and without each factor of interest.

## Results

### Prevalence of *Dirofilaria repens* and factors influencing prevalence in dogs

The DNA of *D. repens* was detected in 59 of 424 examined dogs (overall prevalence 13.9%).

Prevalence was almost twice as high in males (17.6%) compared with females (9.2%) (host sex × *D. repens* infection: χ^2^_1_ = 6.35, *P* = 0.012). Dogs infected with *D. repens* were found in six out of eight countries, including dogs from three Baltic States (Table 1). Differences in prevalence between dogs from different countries were significant (country × *D. repens* infection: χ^2^_7_ = 57.9, *P* < 0.001). The highest prevalence was recorded in sled dogs from Lithuania and Belarus, lower in sled dogs from Latvia and Poland, and only one sled dog from Estonia was found to be positive (Table 1). No *D. repens* infections were detected among the few dogs tested from Finland and Russia, but six pet dogs from the Ukraine tested positive (3.8%).

There was also a significant difference between years of study (year × *D. repens* infection: χ^2^_1_ = 9.54, *P* = 0.002), reflecting mainly a three-fold reduction in prevalence between 2017 and 2019 in sled dogs from Lithuania and Latvia (Table 1). Among Polish sled dogs prevalence was almost identical in 2017 and 2019 (Table 1). Overall prevalence in dogs in 2017 was 39.4% but only 8.8% in 2019 when large numbers of non-sled dogs were tested. Since there was a highly significant difference in prevalence of *D. repens* between sled dogs (four-fold higher) and other dogs (mainly pets; dog status × *D. repens* infection: χ^2^_1_ = 23.0, *P* < 0.001; Table 1), this also contributed to the lower overall prevalence in 2019.

As expected, prevalence was lowest in age class 1 (9.2%) and higher in age classes 2 and 3 (15.0 and 15.5%, respectively) but these differences were not significant when other factors had been taken into account (age class × *D. repens* infection: χ^2^_2_ = 3.80, *P* = 0.15).

Interestingly, among sled dogs from Estonia (Parnau region) ranging in age from 11 months to 14 years, *D. repens* DNA and microfilariae in blood smear were detected solely in the youngest dog (11 months old). An interview with the owner of the positive dog revealed that this dog had been purchased at age of six months and imported from Lithuania (Vilnius region), where two kennels that were most affected by *D. repens* were identified (description below). Thus, in the absence of infection among the other dogs in this kennel and known long pre-patent period this case is clearly attributable to importation from Lithuania.

Two large (20–30 dogs) sled dog kennels from Lithuania (Vilnius region) were sampled both in 2017 and 2019, providing an opportunity to compare both changes in overall prevalence of infection and tracking of individual dogs. At the first sampling in summer-autumn 2017, the owners showed no awareness of this vector-borne parasite and had taken no action to prevent/monitor infection status. In both kennels, a very high prevalence of *D. repens* infection was detected (kennel A: 45.5%; kennel B: 80%) at that time by PCR (12S rDNA). Moreover, in all five positive dogs from the first kennel, the microscopic observation of blood smears revealed the presence of microfilariae, including three smears with numerous larvae. Following our identification of *D. repens* in these kennels in 2017, the owners of both kennels implemented treatment with oral administration of ivermectin at a dose of 6 mcg/kg/month in at least three monthly repeated treatments as recommended in Plumb’s Veterinary Drug Handbook^45^. In kennel B, short-lasting adverse side effects were observed in two dogs following the first ivermectin administration, but were not observed again when treatment was repeated. In both kennels, an approximately three-fold reduction in prevalence of *D. repens* was recorded in 2019 (prevalence in kennel A: 16.7%; in kennel B: 28.6%).

Sixteen dogs from these two kennels were sampled both in 2017 and 2019. In 2017, ten of these dogs tested positive and six negative for *D. repens*. Infected dogs were treated as described above. As reported by the owners, administration of Nexgard spectra (afoxolaner and milbemycin; Merial) twice a year or ivermectin treatment three times a year were used as preventive treatment against filariae between 2017 and 2019 in selected dogs in kennel A and in all dogs in kennel B, respectively. In 2019, the second testing revealed that the six negative dogs remained negative (no new infections) and five out of the ten originally positive dogs now tested negative. However, five dogs were positive at the second sampling (success of ivermectin treatment = 50%).

Interestingly, the owner of the most affected kennel B (80% infected dogs in 2017) reported the importation of five adult dogs from the Czech Republic during the last ten years, for breeding and support of racing teams. One of these dogs, a 6-year-old female, was imported in 2012. During our first sampling in 2017, this female tested positive and presented with a small soft nodule on the head (see below). Following treatment, the dog was negative in 2019.

### Reported symptoms

The majority of sled dog owners reported no health problems during interviews at the site of sampling. However, four dog owners, including the owners of the two most affected Lithuanian kennels, an owner from Grodzisk Mazowiecki (3 out of 4 dogs tested positive) and another from Błędowo (both in Central Poland) reported some symptoms: skin problems (skin lesions, poor quality of fur in Siberian huskies, itching) in kennel A in 2017; small soft nodules in subcutaneous tissues in three dogs in kennel B and in one dog from Błędowo; and stamina problems in dogs from Grodzisk Mazowiecki. All dogs with recorded nodules tested positive for *D. repens* in subsequent PCR testing.

### Microscopy

Microscopic observation of Hemacolor-stained blood smears revealed the presence of microfilariae in nine slides (9/168 = 5.36%). Microfilariae were found in five dogs from Lithuania (57 slides examined), in three dogs from Poland (77 slides examined), in a single positive dog from Estonia (10 slides examined) but were not found among 24 slides from Latvia, including two dogs positive by PCR.

### Molecular identification of *D. repens*

To confirm the specific detection of *D. repens* using the three primer sets detailed above*,* PCR products from 36 positive dogs (61% of the 59 positive samples) were sequenced, representing six countries (15 from Lithuania, 6 from Latvia, one from Estonia, 6 from Poland, 6 from Ukraine and two from Belarus). Sequences were aligned and compared with records in the GenBank database. Only *D. repens* DNA was identified in all sequenced samples.

Fourteen 12S rDNA sequences (7 from Lithuania, 4 from Latvia, one from Poland and two from Belarus) displayed the highest identity (96.45–100%) with the sequence of *D. repens* isolated from a human oral case in France (MT012529) and with the sequence of ‘*Dirofilaria repens* voucher 60 Poland’ obtained from the blood of a dog (KX265088)^43^.

Twenty one COI gene sequences (8 from Lithuania, 2 from Latvia, one from Estonia, 5 from Poland and 5 from Ukraine) displayed the highest identity (97–100%) with the sequence of *D. repens* isolated from a human case in Croatia (KX265049) and with the sequence of *D. repens* isolated from a dog skin in Italy (KX265048).

Finally, 20 NAD1 sequences (8 from Lithuania, 2 from Latvia, one from Estonia, 3 from Poland and 6 from Ukraine) displayed the highest identity (97–100%) with the sequence of *D. repens* isolated from dog blood in Hungary (KX265091) and several other sequences from human and canine cases. Lower homology for several sequences was the result of a lower quality outcome of sequencing but was enough to confirm species identification.

### Detection of *D. immitis* antigens

The SNAP test for detection of *D. immitis* antigens was carried out in 2019 on a total of 167 sled dogs from Lithuania (*n* = 46), Latvia (*n* = 24), Estonia (*n* = 20) and Poland (*n* = 35), and for 42 ‘other dogs’ from Poland, including 20 dogs positive for *D. repens* (2019: Table 1). No positive results were obtained and no cross-reaction with *D. repens*-infected dogs was detected. A false positive result was obtained in only one dog despite double testing (all the dots in the SNAP test lit up, including the negative control), but this dog was negative for *D. repens* both by PCR and blood smears.

## Discussion

The main finding of the present study is the high prevalence of *D. repens* infection in sled dogs from Lithuania, Latvia and Poland with the absence of *D. immitis* infection. Prevalence was high in all these countries, thus confirming that they constitute new endemic regions and hence *D. repens* infections appear now to be endemic and hyper endemic in the majority of NE European countries, including the Baltic States. Additionally, occurrence of *D. repens* was confirmed in dogs from Belarus, Ukraine and Estonia.

In Poland, the first cases of dirofilariasis were recognized in 2009 and 2010, and a few years later prevalence in dogs from Central Poland exceeded locally 25–50%^8,9,40,44,46–48^. Since that time, awareness about this parasitosis has increased, resulting in both more testing of dogs and the use of specific chemoprophylaxis. This trend is apparent also in Southern Europe and has lead to a reduction in the prevalence of *D. immitis* in endemic and hyperendemic regions^2^. In the present study, we detected *D. repens* DNA in about 12% of dogs from Poland, thus indicating some degree of reduction in comparison to earlier years^40,44^ and possibly stabilization. Our observations therefore signify that greater awareness of the risk of infection by sled dog owners and veterinary practitioners has helped to control the occurrence of *D. repens* in recent years. Nevertheless, this reduction in prevalence and stabilization has been accompanied by a consistent appearance of new human cases in Poland^22,24,25^.

Although in Lithuania the first cases in dogs and humans were diagnosed also only in 2010 and 2011, respectively^37,38^, the reported prevalence in dogs was much lower (4% in pet dogs and 19% in shelter dogs) and accompanied by much lower owner and veterinarian practitioner awareness (personal observation during sampling in 2017). However, our sampling in 2017 revealed a very high prevalence in sled dogs from the Vilnius region (58.1%), establishing this as a hyperendemic area. Accordingly, in a recent publication several autochthonous human cases were identified in both Kaunas and Vilnius regions of Lithuania^37,38^.

Both human and canine cases of *D. repens* infections have been previously recorded in Latvia, including in the Riga area^37^. Interestingly, all positive samples derived in 2019 and a few of the samples from 2017 in our study also originated from sled dog kennels from the Riga area. Unfortunately, the place of origin of other positive dogs, sampled during sled dog competitions, was not recorded. Prevalence in sled dogs from Latvia was lower than in Lithuania, and accompanied by a higher awareness and knowledge about dirofilariasis among owners (i.e. one person reported previous treatment of a *D. repens* infection in a dog). Thus, the Riga area appears to have been an established focus of *D. repens* transmission in Latvia for several years now^37^.

Prevalence was lowest in Estonia and the single positive dog, from kennel in the vicinity of Parnau city in Central Estonia, was imported from Lithuania at an age of 6 months, probably already infected with *D. repens* (imported case). No other dogs from this kennel (20 dogs) were positive for microfilariae at the time of sampling, however, transmission from the infected dogs to other dogs in this kennel cannot be excluded, as sampling took place at the end of July 2019, following two warm summer months of vector activity. The spread of *D. repens* within large sled dog kennels can be rapid, because high accessibility of canine hosts and the abundance of mosquitoes offer suitable conditions for the development and transmission of filariae. Imported cases of both *D. immitis* and *D. repens* infection have been reported previously in both Baltic and Nordic countries^34,37^. Moreover, the increase in movement of dogs following the introduction of the Pet Travel Scheme in 2000 in the EU is pointed to by many authors as one of the main causes of the spread of *Dirofilaria* spp. in Europe^1–3,11,18^. It is pertinent also that previous papers based on questionnaire studies among veterinary practitioners^34^ or accidental detection of microfilariae in dog blood^37^ have suggested that *D. repens* may have been endemic already in all Baltic States. Three published cases of likely autochthonous *D. repens* infection in dogs from the Tartu area give an overall prevalence of 0.05% for this region of Estonia^37^.

The significance of importation of infected dogs from endemic areas for establishment of new foci is supported also by the history of dog purchases by kennel B from Lithuania. As reported by the owner, this kennel has several adult dogs imported from the Czech Republic, which is a recognized endemic region for *D. repens*^29^. The imported dogs were not tested before arrival. Importantly, following the sampling in 2017 we confirmed *D. repens* infection in the female imported in 2012, so the time/origin of infection cannot be established with certainty. It is likely that these imported dogs served as a source of infection for the local population of vectors and then for local dogs. Moreover, the positive dog from Estonia originated from the same area in which kennel B is located, thus presenting a possible ‘chain’ of translocation of *D. repens*-infected dogs: from Czechia, through Lithuania to Estonia, about 1200 km to the North in just several years.

In Western Ukraine, prevalence of *D. repens* infection was low among pet dogs compared to sled dogs from other countries, but was similar to prevalence detected in ‘other dogs’ in Poland. Pet dogs live often in their owners’ flats/houses and are not exposed to so many mosquito bites as sled dogs living in outdoor kennels. In the Ukraine, dirofilariasis is a notifiable disease and more than 1400 human cases have been reported to date with a notable increase in recent years^14,15,19^.

For three of the countries in our survey sample sizes were limited, but nevertheless our results are informative and shed some light on the status of local dirofilariasis. Three out of five sampled sled dogs from Belarus tested positive. The owner was not aware of the endemic status of Belarus for dirofilariasis and the dogs had not received any prophylactic treatment. In Belarus at least 80 human cases were recorded between 1997 and 2013^20^ and *Dirofilaria* spp. have been found also in mosquitoes^49^.We did not identify *D. repens* infections among 11 dogs from Russia, but prevalence in different regions of Russia differs profoundly, and has been reported to be below 1% in the vicinity of Moscow (reviewed in:^21^). Similarly, we did not find *D. repens* among the six sampled sled dogs from Finland, and to-date only a few cases of *D. repens* infections have been reported from this country, including one probably autochthonous human case in Southern Finland. Two reported cases in dogs were apparently imported from Russia^37^.

Generally, we found that prevalence was higher in males compared to females, and this is consistent with similar observation by other authors^38,46^. Prevalence was also higher in dogs older than two years, which is in accord with the known long prepatent period before microfilariaemia becomes detectable, the long life span of adult filariae in canine hosts (2–5 years)^1,8,40^ and longer duration of exposure of older animals to vector bites.

We did not detect any *D. immitis* infection in dogs sampled in 2019, although *D. immitis* is believed to follow *D. repens* in its expansion in Central and NE Europe^2,10,16,17,34^. However, *D. immitis* infections in sled dogs participating in training and races should be easily noticed by owners due to exercise intolerance, a characteristic consequence of heartworm infection^50^. Hence lack of these symptoms in the dogs we sampled is in agreement with the negative results of our SNAP tests. In a previous study, *D. immitis* infections were reported from the Baltic and Nordic countries but mostly representing imported infections^34^. In Poland, *D. immitis* has been detected to date in just a few dogs^35^. Heartworm is endemic in the Ukraine^1,2,14^, but unfortunately it was not possibleto perform SNAP tests for the dogs we sampled from this country.

Finally, oral administration of ivermectin for the treatment of *D. repens* infection in two Lithuanian kennels was only partially successful, as microfilariae were detected again in 2019 in 50% of the treated dogs that were *D. repens* positive in 2017, although the possibility of re-infection before the second blood test in 2019 cannot be totally excluded. Ivermectin appears to be less effective in the treatment of *D. repens* infection than moxidectin^18^. Thus, the owners of those infected dogs were advised to implement treatment with topical administration of moxidectin 2.5 mg/kg body weight (plus imidacloprid 10 mg/kgbody weight) as recommended by Genchi and Kramer^18^. The absence of new infections and the general reduction in prevalence of infection in sampled dogs, including two highly affected Lithuanian kennels are positive indications for the future from the present study and are at least partially attributable to an increased awareness of dirofilariasis of owners and veterinarian practitioners. Awareness is crucially important in the control of mainly asymptomatic infections in dogs, facilitating rapid implementation of appropriate chemotherapy and thus prevention of spread of dirofilariasis and establishment of new foci of infection.

## Conclusions

Systematic sampling of sled dogs in the Baltic countries revealed a high prevalence and the hyperendemic status of *D. repens* infections in Lithuania and Latvia. *Dirofilaria repens* infections were found in the majority of sampled countries, in contrast to the lack of positive results when screening for *D. immitis*. Awareness of *D. repens* endemicity has helped to reduce prevalence of infection in some countries. Prevalence was also much higher in sled dogs than in other dogs/pet dogs, which is in agreement with previous studies on working dogs^21,32^. Sled dog kennels appear to constitute hotspots for *D. repens* transmission and thus establishment of new foci of infection.

## Supplementary information

Supplementary Information 1.
