# Supplementary material for: Emerging risk of Dirofilaria spp. infection in Northeastern Europe: high prevalence of Dirofilaria repens in sled dog kennels from the Baltic countries
Source: Sci Rep. 2021 Jan 13;11:1068. doi: 10.1038/s41598-020-80208-1 (PMC7806926; doi:10.1038/s41598-020-80208-1)
Supplement: Supplementary file 1 — Supplementary Information 1. [file 41598_2020_80208_MOESM1_ESM.docx]

**Emerging risk of *Dirofilaria* spp. infection in Northeastern Europe: high prevalence of *Dirofilaria repens* in sled dog kennels from the Baltic countries**

Mustafa Alsarraf, Viktoria Levytska, Ewa J. Mierzejewska, Vasyl Poliukhovych, Anna Rodo, Mohammed Alsarraf, Dziyana Kavalevich, Dorota Dwużnik, Jerzy M. Behnke, Anna Bajer

Supplementary Table 1

Structure of samples: by country, breed, sex and age of dogs

| **Country of origin** | **Year** | **Breed** | **Sex** | Age groups | Total number |
| --- | --- | --- | --- | --- | --- |
| Ukraine:  (155) | 2019 | Different breeds (dogs attending vet clinic) | Males 85  Females 70  Total 155 | <2 years: 36  2-8 years: 94  >8 years: 25 | 155 |
| Poland: sled dogs (n=66) | 2017 and 2019 | Alaskan husky: 29  European Sled Dog (ESD): 20  Siberian Husky: 13  Other: 4 | Males 32  Females 34  Total 66 | <2 years: 10  2-8 years: 29  >8 years: 27 | 66 |
| Poland: other breeds (n=42)  Combined | 2019 | Different breeds (healthy): 44 | Males 25  Females 17  Total 42 | <2 years: 9  2-8 years: 22  >8 years: 11 | 42  108 |
| Lithuania: sled dogs | 2017 and 2019 | Alaskan husky: 23  European Sled Dog (ESD): 36  Siberian Husky: 15  Other: 3 | Males 48  Females 29  Total 77 | <2 years: 21  2-8 years: 44  >8 years: 12 | 77 |
| Latvia: sled dogs | 2017 and 2019 | European Sled Dog (ESD): 5  Siberian Husky: 34  Other: 1 | Males 21  Females 19  Total 40 | <2 years: 7  2-8 years: 23  >8 years: 10 | 40 |
| Estonia: sled dogs | 2017 and 2019 | Alaskan husky: 14  Alaskan Malamute: 4  European Sled Dog (ESD): 2  Siberian Husky: 2 | Males 13  Females 9  Total 22 | <2 years: 1  2-8 years: 17  >8 years: 4 | 22 |
| Finland: sled dogs | 2017 and 2019 | European Sled Dog (ESD): 4  Other: 2 | Males 3  Females 3  Total 6 | <2 years: 2  2-8 years: 4 | 6 |
| Russia: sled dogs | 2019 | Alaskan husky: 2  European Sled Dog (ESD): 4  Siberian Husky: 5 | Males 7  Females 4  Total 11 | <2 years: 1  2-8 years: 9  >8 years: 1 | 11 |
| Bielarus | 2017 | Alaskan husky: 5 | Males 5 | 2-8 years: 5 | 5 |
| Total sled dogs | 2017 and 2019 | Alaskan husky: **73**  Alaskan Malamute: **4**  European Sled Dog (ESD): **71**  Siberian Husky: **69**  Other: **10** | Males 129  Females 98  Total 227 | <2 years: 42  2-8 years: 131  >8 years: 54 | 227 |
| Total other | 2017 and 2019 | Different breeds: **197** | Males 110  Females 87  Total 197 | 2 years: 45  2-8 years: 116  >8 years: 36 | 197 |
| Overall total | 2017 and 2019 | All breeds | Males 239  Females 185  Total 424 | 2 years: 87  2-8 years: 247  >8 years: 90 | 424 |
